# Pancrelipase as Adjunctive Therapy in Severe SCOT Deficiency: A Case of a Novel OXCT1 Gene Deletion

**DOI:** 10.1002/jmd2.70024

**Published:** 2025-05-22

**Authors:** Mo'ath Abu Hamdeh, Lema Jaber, Jamal Abdullah, Anas Manhal, Mahmoud M. Qouqas, Mohammed Aldwaik, Sarah Abu Rmeilah, Mutaz Sultan, Shaher Shweiki, Nadirah Damseh

**Affiliations:** ^1^ Faculty of Medicine Hebron University Hebron Palestine; ^2^ Princess Alia Governmental Hospital Hebron Palestine; ^3^ Department of Pediatrics and Genetics Al Makassed Hospital Jerusalem Palestine; ^4^ Faculty of Medicine Al‐Quds University Jerusalem Palestine

**Keywords:** ketonuria, metabolic acidosis, OXCT1 gene, SCOT, succinyl‐CoA:3‐ketoacid‐CoA transferase

## Abstract

Succinyl‐CoA: 3‐oxoacid CoA transferase (SCOT) deficiency is a rare autosomal recessive disorder caused by biallelic sequence variants in the *OXCT1* gene. This deficiency disrupts ketone body utilization, resulting in ketone accumulation and ketoacidosis. Clinical manifestations typically include respiratory distress, vomiting, lethargy, and, in severe cases, coma. This case presents the first known instance of severe SCOT deficiency resulting from a novel homozygous four‐exon deletion (exons 4–7) in the OXCT1 gene. The proband presented at the age of 3 months with severe metabolic acidosis that was refractory to conventional management. Despite high doses of bicarbonate therapy and cornstarch, he remained dependent on intravenous glucose for weeks. Repeated attempts to discontinue intravenous glucose led to severe acidosis within 12–24 h. The introduction of pancreatic enzyme replacement therapy (Creon) significantly enhanced starch digestion and absorption, stabilizing his metabolic condition and enabling discharge within 3 days. This case highlights the therapeutic potential of combining pancreatic enzyme replacement with cornstarch in infants under 12 months of age, given their limited pancreatic amylase activity. It underscores a potential management strategy for infants with severe forms of inherited metabolic disorders, such as SCOT deficiency and glycogen storage disease type I, where cornstarch is a cornerstone of therapy.

1

**Summary**

- This report documents the first known instance of severe SCOT deficiency due to a homozygous deletion of exons 4–7 in the OXCT1 gene, expanding the genotypic spectrum of this rare disorder.
- The successful combination of pancreatic enzyme replacement (Creon) with cornstarch therapy overcame the challenge of immature pancreatic amylase in an infant, helped achieving metabolic stability.
- This case highlights the potential of combining cornstarch with pancreatic enzymes as a therapeutic strategy in infants with SCOT deficiency or similar metabolic conditions where carbohydrate utilization is essential for disease control.

## 2 Introduction

Mitochondria in the liver are the primary site of ketogenesis but do not express the SCOT enzyme. As a result, ketones produced in the liver are exported to extrahepatic tissues, including the brain, where they are metabolized. Ketone bodies serve as a critical alternative energy source during periods of limited glucose availability, such as fasting or prolonged exertion, accounting for nearly two‐thirds of the brain's energy demand in these states. SCOT is essential for ketone body metabolism, catalyzing the conversion of acetoacetate to acetoacetyl‐CoA, which is then split into acetyl‐CoA for entry into the tricarboxylic acid cycle, generating ATP [1].

SCOT deficiency, an autosomal recessive disorder caused by bi‐allelic sequence variants in the *OXCT1* gene (contains 17 exons), impairs the use of ketone bodies, leading to their accumulation and subsequent ketoacidosis. Symptoms of ketoacidosis vary but typically include respiratory distress, loss of appetite, vomiting, lethargy, and in severe cases, loss of consciousness and coma. Around 61 cases have been reported, with approximately 41 sequence variants in the *OXCT1* gene identified, including missense (29 variants), nonsense (6 variants), intronic (3 variants), insertion (two variants), and exon deletion (one variant) [2, 3, 4, 5, 6]. Early onset occurs in 70% of cases, with most presenting within the first year of life. Without prompt diagnosis and treatment, the condition carries a high risk of fatality [2].

SCOT deficiency is diagnosed through enzyme assays and genetic testing, often after excluding other causes of acidosis. There is no established consensus on management; however, it generally involves administration of carbohydrate, avoiding prolonged fasting and excessive fat intake, mildly restricting protein intake to reduce ketogenesis, and sometimes accompanied by carnitine supplementation. Acute episodes of severe metabolic acidosis as well as persistent metabolic acidosis might be treated with sodium bicarbonate [2, 3].

We present a case of severe SCOT deficiency caused by a novel bi‐allelic deletion in the *OXCT1* gene. Despite the implementation of previously described therapeutic interventions, including high‐dose cornstarch administration, the infant remained reliant on intravenous glucose, likely due to insufficient pancreatic amylase activity. This was successfully managed with pancreatic enzyme replacement therapy. Here, we present the first case in the literature with bi‐allelic homozygous deletion in the *OXCT1* gene. Additionally, we introduce a potential management strategy for a challenging severe case of SCOT deficiency in a young infant.

## 3 Case Presentation

A 3‐month‐old male, the first child of consanguineous Palestinian parents, had an unremarkable antenatal and perinatal history. The infant was delivered via cesarean section at 40+ weeks of gestation due to fetal distress, with a birth weight of 3200 g. At 3 months of age, he presented with respiratory distress, hypotonia, and hypoactivity following a viral illness. Initial laboratory tests were remarkable for severe metabolic acidosis (pH 6.7, HCO_3_^−^ 3 mmol/L, PCO2 16.7 mmHg). He was diagnosed with bronchiolitis and dehydration. He was urgently transferred to the pediatric intensive care unit (PICU) for intubation and mechanical ventilation. He was managed with fluid resuscitation, nebulizers, and antibiotics. After 2 days, he was weaned off the ventilator and transferred to the general pediatric ward. Comprehensive laboratory assessments were performed to evaluate the metabolic disturbance. Blood gas analysis revealed severe acidosis, with subsequent tests showing gradual improvement following intravenous fluids and sodium bicarbonate administration. The pH ranged from 7.14 to 7.38, HCO_3_^−^ levels fluctuated between 3 and 15.5 mmol/L, and PCO2 varied between 12 and 26 mmHg. Ammonium levels were initially elevated at 99 μmol/L but subsequently normalized to 34 μmol/L. Lactate levels remained normal (< 2 mmol/L). Glucose levels were mostly normal, except for one reading of 54 mg/dL. Plasma amino acids were within normal limits, while urine organic acid analysis revealed substantial ketone excretion, including 3‐hydroxybutyrate and acetoacetate. Despite adequate hydration and feeding, the patient persistently tested positive for urine ketones (+1 to +3). Urea and creatinine levels were within normal limits. Both abdominal ultrasound and echocardiogram were unremarkable.

Given the clinical and laboratory findings, the patient was suspected to have a ketolytic disorder, specifically Succinyl‐CoA: 3‐oxoacid CoA transferase (SCOT) deficiency. Initial management included oral L‐carnitine (100 mg/kg/day), sodium bicarbonate (up to 10 meq/kg/day), and careful fluid management, which led to stabilization. However, the patient continued to develop severe metabolic acidosis within 12–24 h of discontinuing intravenous glucose fluids, despite high doses of sodium bicarbonate. The patient was subsequently transferred to a tertiary care center for further management, where exclusive breastfeeding was maintained, and uncooked cornstarch was introduced. The dose of cornstarch was gradually increased to 110 g/day, divided into six meals (3 g/kg/meal). Sodium bicarbonate was administered at a dose of 6.5 meq/kg/day in four divided doses. Despite these efforts, intravenous fluids could not be entirely discontinued.

To address the persistent need for glucose, Glycosade (modified cornstarch) was introduced at 30 g twice daily (12 a.m., 12 p.m.), which allowed a reduction in cornstarch meals to two per day (6 a.m., 6 p.m.). However, intravenous fluids could not be completely discontinued, and multiple attempts to wean off intravenous glucose over a two‐week period were unsuccessful.

Given the immature amylase function in infants, which is essential for the digestion of complex carbohydrates such as cornstarch, we introduced pancreatic enzyme replacement therapy (Creon) 5000 IU with each cornstarch meal. This adjustment, which was initiated at around 4 months of age while the patient was still hospitalized, led to stabilization of blood gas values (pH 7.44, PCO2 31 mmHg, HCO_3_^−^ 22.8 mmol/L) and allowed discontinuation of intravenous glucose within 3 days. The patient was eventually discharged on the same doses of cornstarch/modified cornstarch, with a reduced sodium bicarbonate dose of 2 meq/kg/day, divided into two doses.

The patient had two additional metabolic decompensations at 6 and 7 months of age, which were successfully managed with glucose intravenous fluids and minor adjustments to his treatment regimen. At 12 months of age (weight: 9.2 kg), the patient's management plan included sodium bicarbonate 10 meq every 8 h (3.2 meq/kg/day), Glycosade 30 g three times daily (8 a.m., 4 p.m., 12 p.m.), and cornstarch 2 teaspoons (1.95 g/kg/meal) three times daily (12 a.m., 8 p.m., 4 a.m.). Follow‐up blood gas results at the outpatient clinic showed a pH of 7.40, HCO_3_^−^ of 19.8 mmol/L, and urine ketones of +2. Pancreatic enzyme replacement therapy was discontinued at age 11 months, and disease control has been stable since that time.

Whole‐exome sequencing (WES) revealed a homozygous 10 881‐bp deletion (chr5:g.41842776_41853656del, GRCh38; NM_000436.4) spanning exons 4–7 of the *OXCT1* gene, which was subsequently confirmed through targeted genetic testing using quantitative polymerase chain reaction (qPCR). Both parents were confirmed as heterozygous carriers of the deletion. Notably, both parents were healthy and had no prior history of metabolic acidosis.

## 4 Discussion

Succinyl‐CoA: 3‐oxoacid CoA transferase (SCOT) deficiency is an exceedingly rare autosomal recessive metabolic disorder. To date, over 40 sequence variants in the *OXCT1* gene have been documented in the literature. This report introduces a novel homozygous four‐exon deletion (exons 4–7) in the *OXCT1* gene, representing the first case of this specific mutation. The only previously reported *OXCT1* deletion involved a 12‐month‐old girl with severe SCOT deficiency, presenting with life‐threatening ketoacidosis requiring renal replacement therapy, caused by compound heterozygous variants (c.1118T>G, p.Ile373Ser) and a large deletion spanning exons (8–16) [7]. Our proband provides additional insight into genotype–phenotype correlations, suggesting that deletions in the *OXCT1* gene may be associated with severe clinical manifestations, including early‐onset disease and recurrent, profound ketoacidosis. While this finding adds to our understanding of the molecular basis of SCOT deficiency, further studies are required to confirm these observations and refine the genotype–phenotype relationship.

There are currently no established clinical guidelines or standardized management protocols for SCOTdeficiency. Management strategies that have been employed include the avoidance of prolonged fasting, administration of uncooked cornstarch to prevent catabolic states, implementation of a mildly protein‐restricted diet, restriction of fat intake. The use of L‐carnitine has been reported in the literature but its efficacy is not proven. Additionally, oral bicarbonate has been used mainly for attenuating ketoacidosis [2, 3].

While conventional treatments, such as cornstarch, are essential, limitations arise in infants under 12 months due to immature amylase activity, impairing the digestion and absorption of complex carbohydrates [8]. Pancreatic enzyme replacement therapy (Creon), a pancreatic enzyme replacement containing amylase, lipase, and protease, is indicated for exocrine pancreatic insufficiency in conditions like cystic fibrosis, chronic pancreatitis, or post‐surgery to aid digestion and nutrient absorption [9]. In our case, the inclusion of pancrelipase as adjunctive therapy resulted in improved metabolic control, stabilization of blood gas levels, and timely discharge.

This case provides insights into the management of severe SCOT deficiency, particularly in infants, where the combination of pancreatic enzyme replacement therapy and cornstarch may offer therapeutic benefits due to limited pancreatic amylase activity at this age. This approach may also hold potential as a management strategy for infants with other severe inherited metabolic disorders, such as glycogen storage disease type I, where cornstarch constitutes a cornerstone of therapy. Further research is warranted to validate this strategy and assess its broader clinical applicability.

## Ethics Statement

This study was approved by the Research Ethics Committee at Al‐Makassed Charitable Hospital.

## Consent

Written informed consent was obtained from the parents.

## Conflicts of Interest

The authors declare no conflicts of interest.

## Data Availability

The data supporting the findings of this study are available from the corresponding author upon reasonable request.
